# The establishment, development and future of the Chinese environmental mutagen society

**DOI:** 10.1186/s41021-021-00232-z

**Published:** 2022-01-24

**Authors:** Jia Cao

**Affiliations:** Toxicology Institute, Preventive Medical College, Army Medical University, Chongqing, 400038 People’s Republic of China

**Keywords:** Chinese environmental mutagen Society (CEMS), International Association of Environmental Mutagen Societies (IAEMS), Asian Association of Environmental Mutagens Societies (AAEMS), Carcinogenesis, Teratogenesis, Mutagenesis

## Abstract

It has been 40 years since the Chinese Environmental Mutagen Society (CEMS) was established in 1981. Now, it has grown a first-level national society in China, which has 15 professional committees and more than 5000 members. Over the past 40 years, the CEMS has been making many contributions to advance the research of environmental mutagens in China and cultivate professional talents in this field. In the twenty-first century, looking back on what the CEMS has gone through and accomplished, and in light of the major changes in our tasks and mission in the new era, we must plan well for the future, to overcome our shortcomings, to embrace greater development of the CEMS.

Since its establishment in 1981, the Chinese Environmental Mutagens Society (CEMS) has just gone through 40 years [1]. Now, the CEMS has grown into a huge body which has 15 professional committees and more than 5000 members and become into a first-level national association in China. As the President of the CEMS, I would like to thank Genes and Environment Journal to edit the Chinese special issue, to introduce environmental mutagen research works in China. As the first word of this issue, it is a good opportunity for reviewing the way of CEMS had already gone, how we face the major challenges in tasks and missions in the new era, how we plan for the future and usher in greater development.

## Period of establishment development (1981–2000)

In the early 1970s, the international academic research community started paying attention to the detrimental effects of environmental genotoxins leading to increased genetic mutations and increased risk of teratogenesis and carcinogenesis. The environmental mutagen societies in the western countries were gradually established and developed in this period. With the International Association of Environmental Mutagen Societies (IAEMS) established in the middle of 1970s, the research of environmental factors on genetic damage started to flourish.

In the 3rd International Conference on Environmental Mutagens held in Tokyo, Japan in September 1981, Dr. Hollaender, the initiator of International Association of Environmental Mutagen Societies (IAEMS) made a request to the President of the Chinese Genetics Society, Dr. Jiazhen Tan, to hold a seminar on environmental mutagens in China. In May 1983, the International Symposium of Environmental Mutagenesis, Carcinogenesis and Teratogenesis was held in Shanghai, and the “Environmental Mutagen Committee of the Chinese Genetics Society” was unanimously approved and established [2]. Dr. Jiazheng Tan (Fudan University, member of the Chinese Academy of Sciences) served as the first president on the committee. In 1988, the Chinese Environmental Mutagen Society (CEMS) was officially established, and became a national society affiliated with the Chinese Association of Science and Technology in 1991(http://www.cnems.org.cn/).

At the founding of the Society, it was determined that a national academic conference be held every 2 years, and this academic tradition has been upheld ever since. In the meantime, four professional committees were set up, and academic activities were conducted focused on their respective topics. They were:

1. Professional Committee on Mutagenesis,
2. Professional Committee on Teratogenesis,
3. Professional Committee on Carcinogenesis,
4. Professional Committee on Anti-Mutagenesis and Anti-Carcinogenesis.

**Fig. 1:**
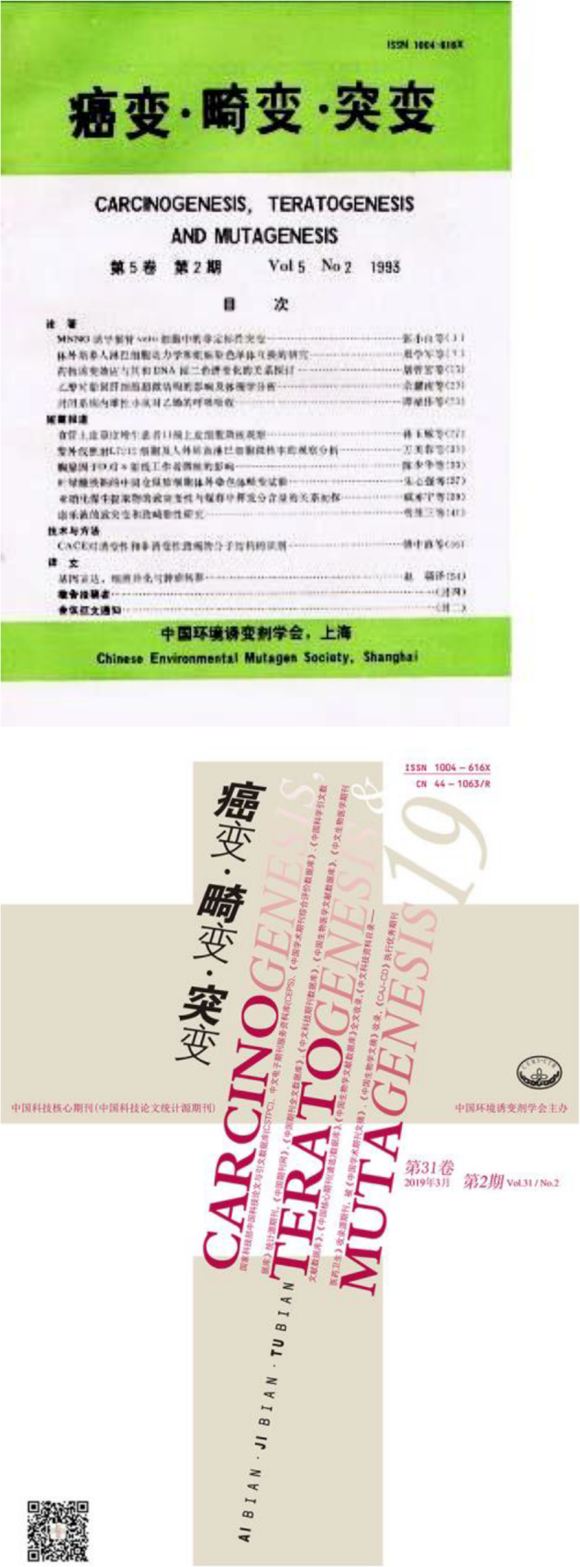
CEMS’s journal entitled “Mutagenesis, Carcinogenesis and Teratogenesis” (Chinese-language, bi-monthly), which has been distributed nationwide ever since in 1991 until now

In order to provide a platform for scientific research and academic exchanges, our society initiated a Chinese-language journal entitled “Mutagenesis, Carcinogenesis and Teratogenesis” (bi-monthly) in 1991 (Fig. 1), which has been distributed nationwide ever since (https://www.egh.net.cn/CN/1004-616X/home.shtml).

During this period, for many Chinese researchers, the study of environmental mutagens, including both theory and methodology, was relatively new, and they could not only discover their own new scientific research directions, but also quickly carry out screening, identification and evaluation of the environmental pollutants which were around their local areas or special occupational exposures. Therefore, the number of CEMS expanded to more than 2000 over this period, and enthusiasm for research was booming. Like many countries in the world, it was a golden period for the environmental mutagen research in China.

Over this period, the CEMS predominantly focused on the environmental damage to genetic material - genetic toxicity detection and evaluation. Major work included:

1. Establishing methodologies: a series of methodologies of classic genetic toxicology were introduced from abroad, such as the Ames test, micronucleus experiments, HPRT gene mutation assay, and so on. We gradually set up relevant laboratories in China and started training, research and outreach in this area.
2. Performing safety evaluation: A large number of environmental chemicals and genetic toxicants that Chinese people had been exposed to were screened and evaluated. In the 1990s, the detection methods for genotoxicity gradually became standardized in China.
3. Making regulatory rules: Nationally, various genotoxicity assays were formally included in all relevant regulations for safety evaluation and testing, making important contributions to the country’s economic and social development.
4. Studying damage mechanism: Some laboratories and scientists started to conduct and strengthen research on the environmental mutagenic mechanisms.

Over this period, with the joint efforts of the older generation scientists and the core members of the CEMS, China made remarkable achievements in the detection methods and related mechanistic research on carcinogenicity, teratogenicity and mutagenicity, with a lot of innovative research, some of which was recognized internationally.

Examples include the following [3–7]:

1. Basic research and clinical trials of recombinant AAV-2/human coagulation factor IX for hemophilia B gene therapy
2. Mutagenesis detection system of transgenic mice carrying the XYLE target gene
3. A series of studies on the association between mutagenic detection and tumors
4. Studies on nontargeted mutations and their molecular mechanisms
5. The in vitro repackaging system of bacteriophage lambda containing the lacZ gene used for the detection of mutations
6. Rodent whole embryo culture system and its applications
7. The anti-mutagenic effects of tea and their related mechanisms
8. The anti-cancer and anti-mutagenic effects and mechanisms of natural foods and fruits.

## Period of steady development (2001–2016)

Since the dawn of the twenty-first century, the development of life science and medicine in the world has accelerated and the overall research quality has greatly improved. The following trends of environmental mutagen research emerged in the field:

1. From the main direction of short-term tests to evaluate mutagenesis and carcinogenesis, transfer to conduct more mechanistic research with the new molecular biology methods.
2. Movement towards using multi-omics technologies, reporter genes, computational toxicology and informatics, bioreactor or biosensor technology to develop new mutation detection methods and biomarkers.
3. Long-term, low-dose combinatorial exposure starting to receive more attention.
4. Population exposure and cohort studies attracting more attention.
5. More attention shifted towards toxicity testing strategies, prediction methods, and safety evaluation models, in order to translate research results into practical use more swiftly.

During this period, environmental mutagen research in China encountered bigger difficulties. The use of traditional and classic genetic toxicity detection methods was gradually expanded in China, but it was difficult to make a breakthrough regarding new methodologies, and mechanistic research was also not thorough enough. In regard to the new methodologies of genetic toxicity detection and the mechanisms of genotoxic effects, the gap between China and the international advances widened. But in the applied research area, China still made a number of characteristic achievements. For example [8–13]:

1. Population studies of the interaction between SNPs and genes,
2. Basic and applied studies on the effects of environmental and genetic factors on male reproductive function,
3. Studies of pollution by persistent toxic substances in water of the Three Gorges reservoir and their health effects.

Over this period, many government officials and scholars found the name and concept of “environmental mutagen” difficult to understand and detrimental to further advocacy and promotion of the academic influence of this field. To this end, from 2003 to 2010, the Chinese Environmental Mutagen Society repeatedly considered changing its name, having considered names such as Chinese Genetics-Environment-Health Society, Chinese Environment and Gene Society, and Chinese Environmental Mutagen and Genomics Society (before the international societies changed their names). However, considering that the Chinese Environmental Sciences Society and the Chinese Genetics Society already had similar professional committees to the CEMS, showing a large crossover and overlap, the name change was not formally implemented.

Over this period, several new professional committees were founded successively in the Chinese Environmental Mutagen Society. They were as follows:

1. Professional Committee on Risk Assessment,
2. Professional Committee on Birth Defects Prevention,
3. Professional Committee on Toxicity Testing and Alternative Methods,
4. Professional Committee on Diet and Disease,
5. Professional Committee on Biological Effects of Reactive Oxygen Species.

This led to the expansion of the CEMS, the number of expert committees increased to 9, and the number of members expanded to more than 4000.

**Fig. 2:**
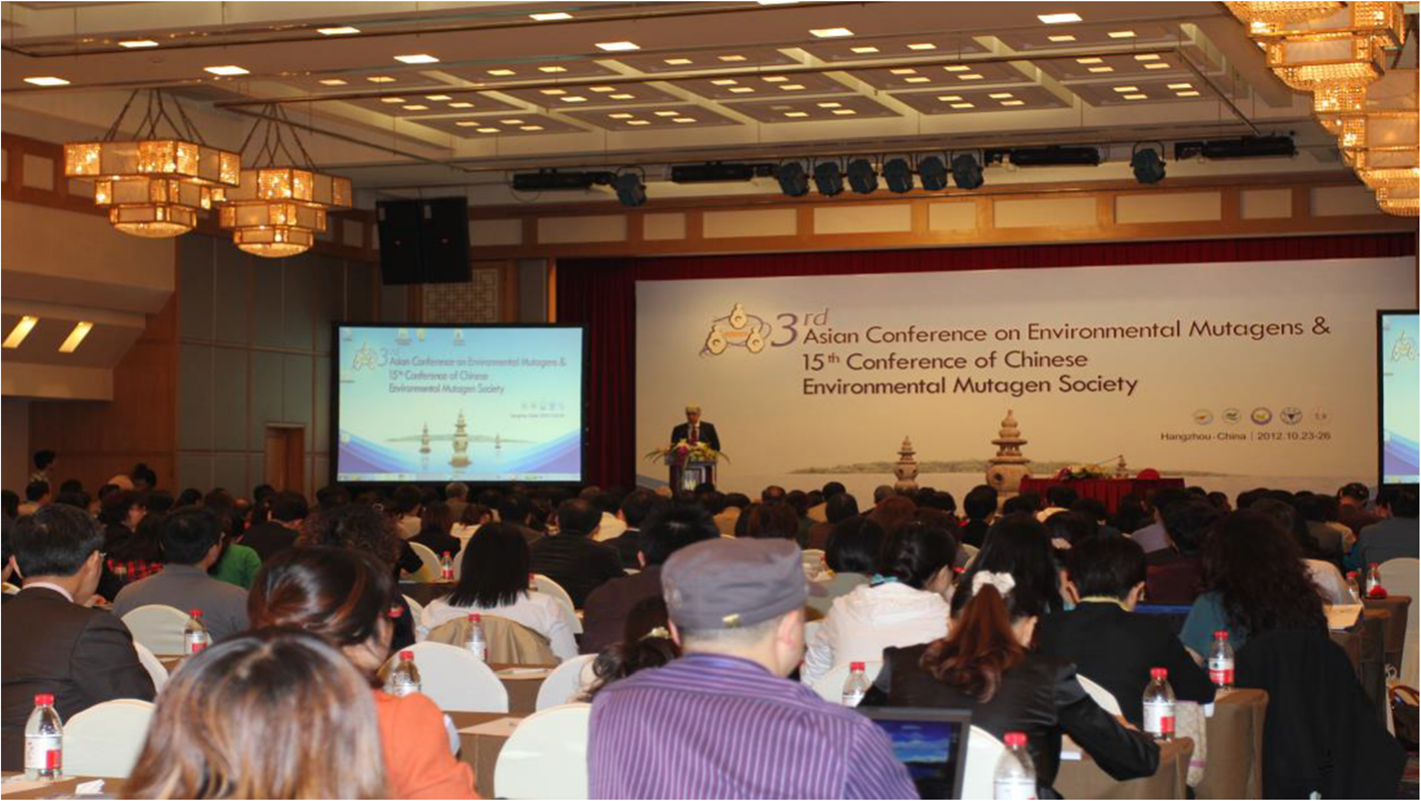
the CEMS hosted the “3rd ACEM Conference and the 15th CEMS National Academic Conference” in Hangzhou, China on 23–26, Oct. 2012

During this period, the CEMS actively participated in all conferences and events of AAEMS in Asia. In October 2012, the CEMS hosted the “3^rd^ ACEM Conference and the 15^th^ CEMS National Academic Conference” in Hangzhou, China, Over 50 foreigner experts from 12 countries and more than 350 domestic participants gathered together for a successful academic conference (Fig. 2) [14].

## Period of new development (2017-)

In recent years, China’s further economic and social development has brought forth higher demands on the health of the whole population. Both domestic and abroad, the rapid development of medical science and technology marked by artificial intelligence (AI), big data, new materials, precision medicine, etc., has brought about greater responsibilities for our environmental mutagen researchers. In December 2017, the 17th National Conference of CEMS was held in Shanghai [15]. The conference proposed that the Society should stand higher and take more proactive measures to keep up with the pace of our time, meet the challenges of environmental mutagen research and make new academic contributions in the new era.

Considering the new frontiers of the field in recent years, a number of new professional committees have been set up recently:

1. Professional Committee on Biomarkers (2018),
2. Professional Committee on Exposure-omics and Exposure Science (2019),
3. Professional Committee on Environment and Reproductive Health (2020),
4. Professional Committee on Environment and Neurodegenerative Diseases (2020),
5. Professional Committee on Environmental Stress Response and Health Impairment (2020).

Young scientists are the vitality and source of academic innovation. The CEMS attaches great importance to the training of young talents, and also set up a special youth committee in 2019. Many outstanding young scientists now working in the field of environmental health in Chinese universities, and doctoral researchers who returned from overseas, have joined us. The CEMS aims to build better academic exchange platforms for them and continue to enhance their innovation and research.

Needless to say, there are still many shortcomings of research in environmental mutagens in China through our reflection. The main concern is that more effort should be focused on innovation to improve basic and applied research capability that is relevant to emerging environmental challenges Furthermore, China’s economic development has entered a new stage, leading to higher demands for environmental protection, and how to consider and do research on air pollutants and emerging environmental pollutants will be the new problems that Chinese scientists face. Moreover, how to further enhance academic exchanges with other countries and make Chinese voices heard more at the Asian Society and the International Society is also something we need to further strengthen.

## In conclusion

Since its foundation more than 40 years ago CEMS has made substantial contributions to basic academic and applied research. In the twenty-first century, CEMS would like to move beyond its tethered name, build a broader stage for the interactions between environment, genetics and health, conduct more innovative academic research, and make greater contributions to the global ‘s economic and social development in the future.

## Data Availability

Not applicable.

## Abbreviations

CEMS: Chinese environmental mutagen society
AAEMS: Asian association of environmental mutagens societies
IAEMS: International association of environmental mutagen societies
ACEM: Asian congress on environmental mutagens
SNPs: Single nucleotide polymorphism
AI: Artificial intelligence
